# Examining the Risks of Major Bleeding Events in Older People Using Antithrombotics

**DOI:** 10.1007/s10557-019-06867-z

**Published:** 2019-03-02

**Authors:** Prasad S. Nishtala, Hamish A. Jamieson, H. Carl Hanger, Te-yuan Chyou, Sarah N. Hilmer

**Affiliations:** 1 Department of Pharmacy and Pharmacology, University of Bath, Building 7 West, Bath, Bath, BA2 7AY UK; 2 Department of Medicine, University of Otago, Christchurch, P O Box 4345, Christchurch, New Zealand; 3 School of Pharmacy, University of Otago, Adams Building, 18 Frederick Street, PO Box 56, Dunedin, 9054 New Zealand; 4 Kolling Institute of Medical Research, Royal Shore Hospital and Sydney Medical School, University of Sydney, Sydney, Australia

**Keywords:** Antithrombotics, Anticoagulants, Aspirin, Warfarin, Bleeding, Atrial fibrillation

## Abstract

**Background:**

Real-world evidence for the safety of using antithrombotics in older people with multimorbidity is limited. We investigated the risks of gastrointestinal bleeding (GI-bleeding) and intracranial (IC-bleeding) associated with antithrombotics either as monotherapy, dual antiplatelet therapy (DAPT) or as triple therapy (TT) [DAPT plus anticoagulant] in older individuals aged 65 years and above.

**Methods:**

We identified all individuals, 65 years and above, who had a first-time event of either IC- or GI-bleeding event from the hospital discharge data. We employed a case-crossover design and conditional logistic regression analyses to estimate the adjusted relative risks (ARR) of bleeding.

**Results:**

We found 66,500 individuals with at least one event of IC- or GI-bleeding between 01/01/2005 and 31/12/2014. DAPT use was associated with an increased risk relative to non-use of any antithrombotics in IC-bleeding (ARR = 3.13, 95% CI = [2.64, 3.72]) and GI-bleeding (ARR = 1.34, 95% CI = [1.14, 1.57]). The increased bleeding risk relative to non-use of any antithrombotics was highest with TT use (IC-bleeding, ARR = 17.28, 95% CI = [6.69, 44.61]; GI-bleeding, ARR = 4.85, 95% CI = [1.51, 15.57]).

**Conclusions:**

Using population-level data, we were able to obtain estimates on the bleeding risks associated with antithrombotic agents in older people often excluded from clinical trials because of either age or comorbidities.

**Electronic supplementary material:**

The online version of this article (10.1007/s10557-019-06867-z) contains supplementary material, which is available to authorized users.

## Introduction

Dual antiplatelet therapy (DAPT) combines aspirin and clopidogrel, ticagrelor or other antiplatelet drugs, and is recommended to reduce the risk of thrombotic events in patients with acute coronary syndromes (ACS) or for individuals undergoing percutaneous coronary interventions (PCI) [1–3]. In an individual with a diagnosis of atrial fibrillation (AF), after ACS or PCI, an anticoagulant drug, typically warfarin or one of the direct oral anticoagulants, is used concurrently with DAPT; this combination treatment is typically known as “triple therapy” (TT) [4, 5].

Studies that have examined the risk of bleeding using DAPT and TT regimens have reported mixed findings. Some studies have shown that the concomitant use of warfarin with DAPT increases the risk of bleeding [6–8]. However, others reported no association of an increased risk of bleeding [9, 10]. Older people prescribed DAPT or TT because of a percutaneous intervention and AF are excluded from clinical trials because of comorbidities or extremes of age. Hence, in the absence of clear guidance from clinical trials and head-to-head comparisons, there remains an ongoing research gap on the bleeding risks associated with antithrombotics in older people with multimorbidity. We need reliable population-level evidence for the safety of using antiplatelet, anticoagulants as either monotherapy, DAPT or TT treatments particularly in older people within the context of varying doses and multimorbidity. In this study, we analysed New Zealand’s pharmaceutical information database (Pharms) and national minimum data set (NMDS), to examine the association of the risks of intracranial bleeding (IC-bleeding) and gastrointestinal bleeding (GI-bleeding) with concomitant use of anticoagulants and antiplatelet either as monotherapy, DAPT or TT regimens in older individuals.

## Methods

The Human Ethics Research Committee, University of Otago approved the study (approval number HD 16/077).

### Source of Data

The New Zealand Ministry of Health maintains national collections of prescription use and hospital discharges. Individual records in these national collections include a unique 7-digit alphanumeric identifier, known as the National Health Index (NHI) identifier. The NHI is encrypted in all datasets, but there is only one encrypted version of each NHI that is never changed. Therefore, we were able to link new data with datasets previously extracted. The pharmaceutical information database (Pharms) includes a record of all prescription claims made by community pharmacists funded by Pharmaceutical Management Agency (PHARMAC). The pharmaceutical collections contain patient demographic information including age, sex, ethnicity, deprivation scores and detailed information about drugs dispensed including drug name, dispensing date, formulation type, administration route, dose, weight, frequency, quantity prescribed and quantity dispensed. The National Minimum Dataset (NMDS) is a collection of hospital discharges for inpatients and day patients. The coding of patients’ diagnoses is in accordance with the International Classification of Diseases and Related health problems tenth revision, Australian Modification (ICD-10-AM).

### Study Design

We identified all individuals, 65 and above with a first-time event of either IC- or GI-bleeding, between 01/01/2005 and 31/12/2014. The index date was the first-time event date for the individual with IC- or GI-bleeding after 01/01/2005. We identified bleeding events using the ICD-10-AM (sixth revision)-coded diagnoses for GI- or IC-bleeding (Supplementary Table 1) from the NMDS. In the spirit of Poulson and colleagues [11], we considered the code for traumatic subdural haemorrhage (S065) to capture majority of the SDH cases.

**Fig. 1:**
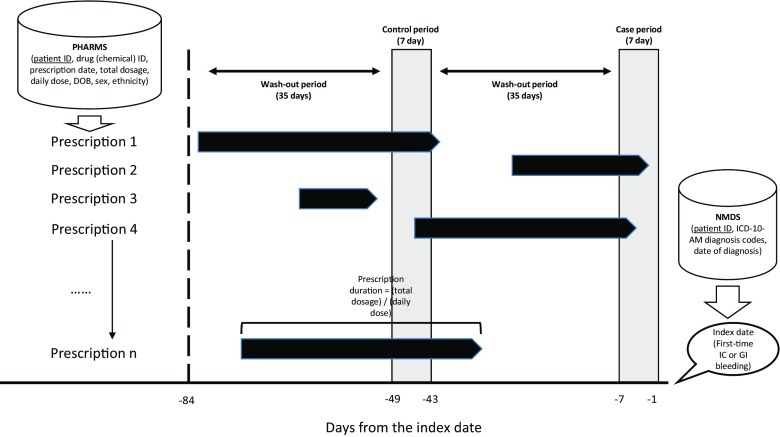
The case-crossover design and the source of data

In this study, we considered only drugs funded by Pharmaceutical Management Agency (PHARMAC) including prescriptions of anticoagulants (warfarin and dabigatran), antiplatelet (dipyridamole, clopidogrel), aspirin and effect-modifiers (Supplementary Table 2). PHARMAC subsided dabigatran from July 2011. In this study, effect-modifiers are drugs that modify the risk of cardiovascular events and haemorrhage. The main effect-modifiers considered in this study included statins (which lowers the risk of a cardiovascular event), selective serotonin receptor inhibitors (SSRIs) (which are associated with increased risk of bleeding) and non-steroidal anti-inflammatory drugs (NSAIDs) (which increase both cardiovascular risk and risk of GI-bleeding) (Supplementary Table 2). We did not consider over-the-counter drugs for this study. We analysed the CSV-formatted datasets using a computer program written in R. Figure 1 depicts the process of case selection as shown. We partitioned the final dataset into an IC-bleeding cohort and a GI-bleeding cohort.

### Statistical Analysis

Analyses were conducted using R version 3.2.4 Revised (2016-03-16 r7033). We used a case-crossover design [12, 13] to examine the population-level association of IC- or GI-bleeding risks associated with concomitant use of anticoagulants, antiplatelet and aspirin (Supplementary Table 2). Two 7-day observation periods with each preceded by a 35-day wash-out period were defined over an 84-day study period (Fig. 1). The case period was the observation period 1–7 days before the index date (day of event). The control period is the observation period 43–49 days before the index date. From the prescription data, we can work out the duration of each prescription by dividing the total dose supplied by the daily dose. Together with the prescription dates, we can determine whether an individual has access to aspirin only (aspirin monotherapy), anticoagulant (AC) only (anticoagulant monotherapy-either warfarin or dabigatran), antiplatelet (AP) only (antiplatelet monotherapy-either dipyridamole or clopidogrel), AP and AC, aspirin and AC, aspirin and AP (DAPT), all three (TT) or none within the case period and the control period. The prescription data do not explicitly indicate whether a particular drug-therapy (i.e. TT, DAPT, AP/AC/aspirin monotherapies or other double-therapies involving AP, AC or aspirin) is taken. Hence, we determined the drug therapies that an individual might be using within an observation period based on the classes of drugs accessible (AP, AC, aspirin or some combinations of them) with the observation period of interest. Table 1 shows the definitions for antithrombotics as monotherapy, DAPT and TT.

**Table 1:**
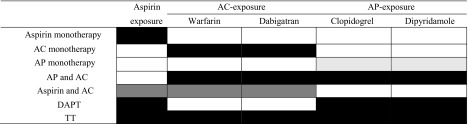
Detailing the definitions of monotherapies and combined therapies

AC-exposure means exposure to warfarin only or dabigatran. AP-exposure means exposure to either clopidogrel or dipyridamole

Time-invariant or slow-changing confounding variables are automatically balanced in a case-crossover design, even if they are unknown [14]. However, the influence of time-variant confounding variables has to be accounted to obtain precise estimates of bleeding risks following antithrombotic exposure. In this study, concomitant use of other drugs that change the risks of cardiovascular events and haemorrhage (i.e. the effect-modifiers) are obvious time-varying confounding variables. From the case-crossover design, for each, we computed the combination of aspirin, AC and AP used over an observation period; and whether or not a bleeding event occurred at the end of the same observation period. Using conditional logistic regression (CLR), we were able to estimate the changed risk of IC- or GI-bleeding associated with monotherapies of aspirin, AC or AP, DAPT and TT as well as other double therapies involving AP, AC or aspirin, relative to non-use. We report the changed risks as relative risks (RR). CLR accounts for the between-individual variation of health characteristics when estimating the changed risk with drug exposure, and thus mitigates confounding. We used multivariable CLR to adjust for the influence of the concomitant use of effect-modifiers (Supplementary Table 2) to calculate the adjusted relative risks (ARR).

## Results

**Fig. 2:**
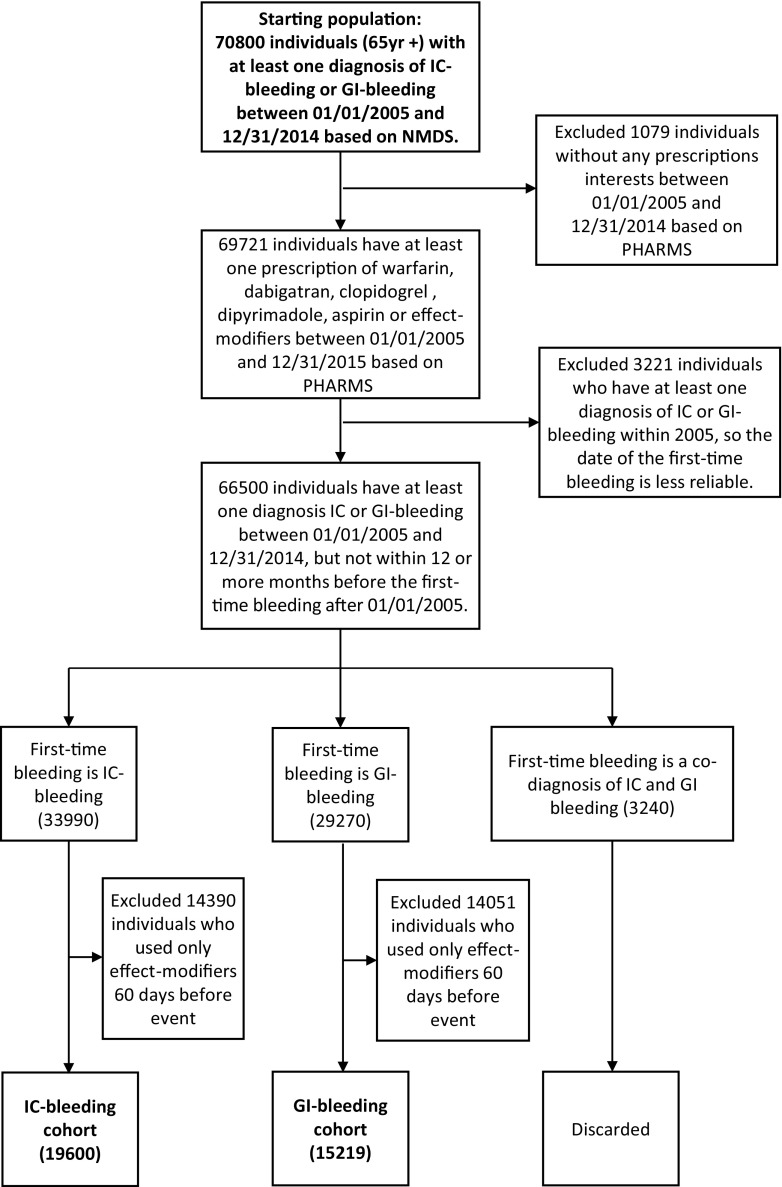
The process of case selection. The length of the study period is 84 days based on Fig. 1

**Fig. 3:**
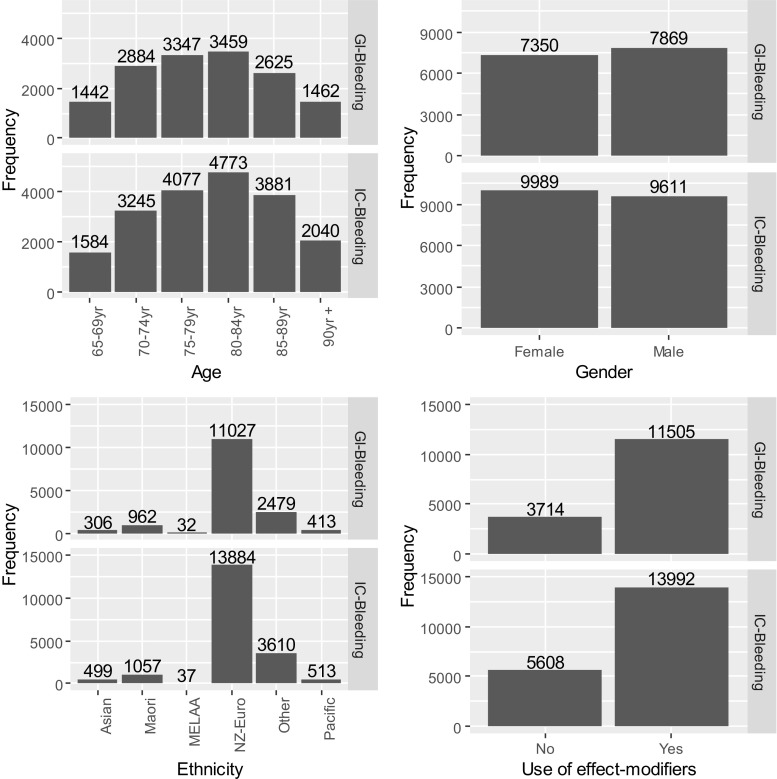
The characteristics of the IC- and GI-bleeding cohorts

We found 70,800 individuals with at least one diagnosis of IC- or GI-bleeding between 01/01/2005 and 31/12/2014 (Fig. 2). We excluded 1079 individuals as they did not receive any prescription of aspirin, AC, AP or any effect-modifiers of interests, and 3221 of them were further excluded because at least one IC- or GI-bleed had occurred within 12 months from 01/01/2005, implying a potentially less reliable date of first-time bleeding (Fig. 2). Amongst the 66,500 individuals remaining, 19,600 of them had IC-bleeding on the index date and received at least one prescription of AC, AP or aspirin 84 days before the event. These individuals were categorised as the IC-bleeding cohort. Furthermore, 15,219 of them had GI-bleeding on the index date and received at least one prescription of the same drugs 84 days before the event, and these individuals formed the GI-bleeding cohort. The characteristics of the study cohorts are shown in Fig. 3. In both study cohorts, males were slightly more than females, and a vast majority of them are NZ-Europeans and only a small proportion of them are Māori (Fig. 3).

**Fig. 4:**
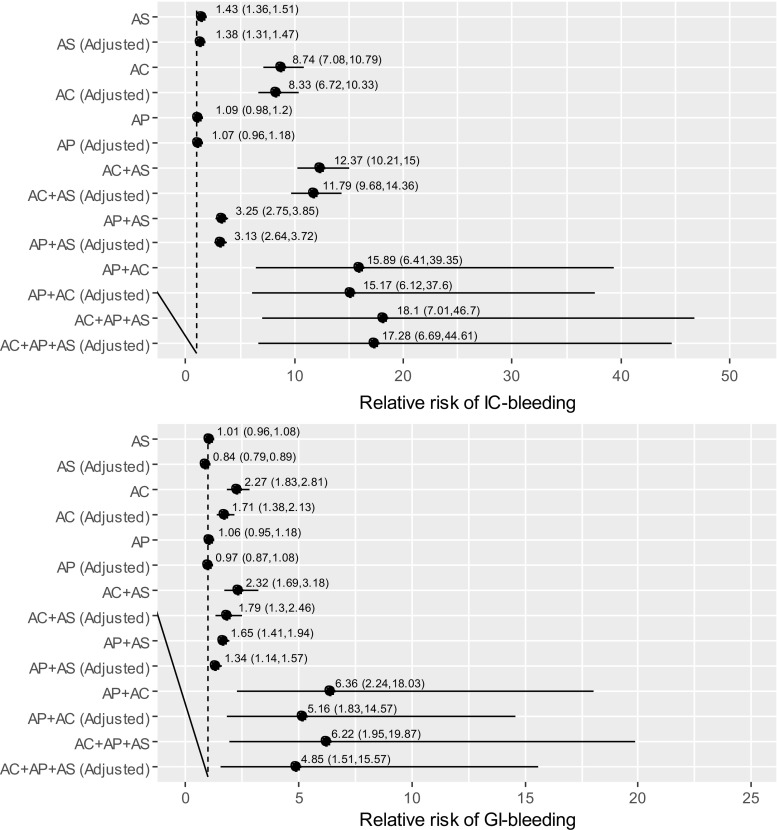
The relative risk of bleeding events associated with the use of poly-therapies involving combinations of aspirin (AS), anticoagulant (AC) or antiplatelet (AP) as well as the monotherapies, relative to non-use of any of these drugs

Figure 4 displays results of the adjusted relative risk (ARR) analysis. Relative to non-use of any antithrombotics, aspirin and antiplatelet monotherapies are associated with a mild increased risk of incident IC-bleeding (aspirin, ARR = 1.38, 95% CI = [1.31, 1.47], AP, ARR = 1.07, 95% CI = [0.98, 1.18]), and no association was found with incident GI-bleeding (aspirin, ARR = 0.84, 95% CI = [0.79, 0.89], AP, ARR = 0.97, 95% CI = [0.87, 1.08]). However, concomitant use of AP and aspirin as DAPT increases the risk of incident IC-bleeding three times comparing to non-use of any antithrombotics (ARR = 3.13, 95% CI = [2.64, 3.72]), and is associated with incident GI-bleeding (ARR = 1.34, 95% CI = [1.14, 1.57]).

The use of anticoagulant monotherapy significantly increases the risk of IC-bleeding (ARR = 8.33, 95% CI = [6.72, 10.33]) relative to non-use of any antithrombotics, and moderately increases the risk of GI-bleeding (ARR = 1.71, 95% CI = [1.38, 2.13]). Despite the wide 95% confidence intervals, the risk of incident IC-bleeding further increases when AC is co-used with aspirin (ARR = 11.79, 95% CI = [9.68, 14.36]) or AP (ARR = 15.89, 95% CI = [6.41, 39.35]) or both aspirin and AP as TT (ARR = 17.28, 95% CI = [6.69, 44.61]) relative to non-use of any antithrombotics. For incident GI-bleeding, the risk further increases when AC is co-used with aspirin (ARR = 1.79, 95% CI = [1.30, 2.46]) or AP (ARR = 6.36, 95% CI = [2.24, 18.03]) or in TT with both aspirin and AP (ARR = 4.85, 95% CI = [1.51, 15.57]) relative to non-use of any antithrombotics.

Overall, the relative risk analysis (Fig. 4) shows that with or without adjusting for concomitant uses of effect-modifiers, concomitant prescribing of aspirin, AP or AC is associated with larger increases in incident IC or GI-bleeding, compared to using them as monotherapies.

## Discussion

This national study found that the use of DAPT and TT regimens is associated with a higher risk of both IC- and GI-bleeding than non-use. Importantly, individuals receiving DAPT (i.e. dual therapy with aspirin and antiplatelet) had a two–three times higher risk of IC-bleeding than individuals on monotherapy, consistent with a cumulative pharmacodynamic effect. A similar effect size of higher bleeding risk was reported in an observational registry-based study conducted in Denmark in individuals (age 73.7 (SD = 12.3)) diagnosed with AF [6]. There is clinical ambiguity surrounding the appropriateness of using combination antithrombotics particularly in individuals diagnosed with AF. Observational studies have unequivocally shown that the combination of warfarin and aspirin is associated with serious bleeding complications; however, the sample sizes in these studies were relatively small compared to our national study. A meta-analysis led by Dentali et al. found an increased risk of bleeding associated with the combination of warfarin and aspirin compared with either, but the heterogeneity of study designs and the variability in the population included limits their external validity. Findings from meta-analyses are not reflective of the real risk posed in high-risk populations such as the elderly given that clinical trials routinely exclude older individuals using DAPT including those with a history of GI ulcers and those taking concomitant drugs.

Interestingly, our study also found an increased risk of IC-bleeding associated with aspirin alone. A systematic review of observational studies found that the overall pooled estimate of the relative risk of IC-bleeding with low-dose aspirin was 1.4 (95% CI, 1.2–1.7). In our study, the adjusted relative risks of IC-bleed associated with aspirin was estimated to be 1.38 (95% CI, 1.31–1.47), similar to the published data.

Triple therapy (TT) regimens are associated with a higher risk of bleeding compared with anticoagulant and aspirin therapy in post-acute myocardial infarction [6]. In a multicenter randomised trial involving 2725 patients with AF who had undergone PCI, the risk of bleeding was lower in patients receiving DAPT compared to TT regimens without any significant differences in thrombotic outcomes [15]. Our analysis found compared to monotherapies that the increased risks of IC- or GI-bleeding associated with DAPT (aspirin and AP) are approximately 1.5–3 times higher after adjusting for concomitant uses of effect-modifiers (i.e. drugs that increase the bleeding risks). Furthermore, the increased risks of IC- or GI-bleeding associated with TT is approximately 6–16 times higher after adjusting for concomitant uses of effect modifiers. Also, combining anticoagulants with DAPT (i.e. turning DAPT into TT) further increases the risks of IC- or GI-bleeding.

The pharmaceutical collections in New Zealand cover more than 95% of the older population, and hence the results are generalizable to the older population. The use of a case-crossover design mitigates confounding from unknown time-invariant confounding variables. The advantage of using case-crossover design is that comparisons are automatically background-matched because they comprise the same individuals, and hence mitigating the influence of unidentified or unmeasured biological and psychosocial confounders.

Our findings are to be interpreted with caution in light of several limitations. A case-crossover design cannot mitigate the effect of unknown time-varying confounding variables. We only adjusted for the concomitant use of effect-modifying drugs as a time-varying confounding variable when calculating the relative risks. Since this study describes observational data from a national collection of hospital events for bleeding, we recommend that several time-varying confounders are to be recognised while interpreting the risks reported in this study such as body mass index and dietary guidance. There is a possibility of channelling bias were doctors could prescribe antithrombotic combinations perceived to be safer in older people, particularly in those they consider to be at higher bleeding risk. Confounding by an indication could potentially limit the validity of our findings. We could not capture bleeding events that did not require hospitalisations, and this could have underestimated the precise bleeding risk associated with antithrombotic therapy. Finally, we could not ascertain if the dispensed medicines were taken before the bleeding event.

Despite the limitations, this national study provides a real-world context and adjusted relative risks of bleeding associated with the use of antithrombotics in older people in the context of multimorbidity. In our future research, we intend to compare the risk of bleeding versus hospital admissions due to acute cardiovascular events to inform the optimal selection of antithrombotics in this vulnerable population.

## Electronic Supplementary Material

ESM 1 (DOCX 15 kb)
