# Supplementary material for: Examining the Risks of Major Bleeding Events in Older People Using Antithrombotics
Source: Cardiovasc Drugs Ther. 2019 Mar 2;33(3):323–9. doi: 10.1007/s10557-019-06867-z (PMC6538582; doi:10.1007/s10557-019-06867-z)
Supplement: Supplementary file 1 — (DOCX 15 kb) [file 10557_2019_6867_MOESM1_ESM.docx]

**Table S1:** ICD-10 AM codes of diabetes diagnosis

| ICD-10-AM | Description | Grouping |
| --- | --- | --- |
| I61 | Nontraumatic intracerebral haemorrhage | IC-bleeding |
| I61.0 | Intracerebral haemorrhage in hemisphere, subcortical | IC-bleeding |
| I61.1 | Intracerebral haemorrhage in hemisphere, cortical | IC-bleeding |
| I61.2 | Intracerebral haemorrhage in hemisphere, unspecified | IC-bleeding |
| I61.3 | Intracerebral haemorrhage in brain stem | IC-bleeding |
| I61.4 | Intracerebral haemorrhage in cerebellum | IC-bleeding |
| I61.5 | Intracerebral haemorrhage, intraventricular | IC-bleeding |
| I61.6 | Intracerebral haemorrhage, multiple localized | IC-bleeding |
| I61.8 | Other intracerebral haemorrhage | IC-bleeding |
| I61.9 | Nontraumatic intracerebral haemorrhage, unspecified | IC-bleeding |
| I62 | Other and unspecified nontraumatic intracranial haemorrhage | IC-bleeding |
| I62.0 | Nontraumatic subdural haemorrhage | IC-bleeding |
| I62.01 | Nontraumatic acute subdural haemorrhage | IC-bleeding |
| I62.02 | Nontraumatic subacute subdural haemorrhage | IC-bleeding |
| I62.03 | Nontraumatic chronic subdural haemorrhage | IC-bleeding |
| I62.1 | Nontraumatic extradural haemorrhage | IC-bleeding |
| I62.9 | Nontraumatic intracranial haemorrhage, unspecified | IC-bleeding |
| S06.5 | Traumatic subdural haemorrhage | IC-Bleeding |
| K92.0 | Hematemesis | GI-bleeding |
| K92.1 | Melena | GI-bleeding |
| K92.2 | Gastrointestinal haemorrhage, unspecified | GI-bleeding |

**Table S2.** Effect modifiers considered in this study.

| Drug name | Drug class | Note |
| --- | --- | --- |
| Warfarin | Anticoagulant | Drug of interest |
| Dabigatran | Anticoagulant | Drug of interest |
| Aspirin | Antiplatelet | Drug of interest |
| Clopidogrel | Antiplatelet | Drug of interest |
| Dipyridamole | Antiplatelet | Drug of interest |
| Omeprazole | Antiulcerants | Effect modifier |
| Omeprazole | Antiulcerants | Effect modifier |
| Omeprazole | Antiulcerants | Effect modifier |
| Pantoprazole | Antiulcerants | Effect modifier |
| Lansoprazole | Antiulcerants | Effect modifier |
| Ibuprofen | NSAID | Effect modifier |
| Naproxen | NSAID | Effect modifier |
| Naproxen | NSAID | Effect modifier |
| Diclofenac | NSAID | Effect modifier |
| Celecoxib | NSAID | Effect modifier |
| Meloxicam | NSAID | Effect modifier |
| Sertraline | SSRI | Effect modifier |
| Citalopram | SSRI | Effect modifier |
| Escitalopram | SSRI | Effect modifier |
| Fluoxetine | SSRI | Effect modifier |
| Paroxetine | SSRI | Effect modifier |
| Mirtazapine | Antidepressant | Effect modifier |
| Mirtazapine | Antidepressant | Effect modifier |
| Phenytoin | Antiepileptic | Effect modifier |
| Carbamazepine | Antiepileptic | Effect modifier |
| Prednisone | Corticosteroid | Effect modifier |
| Prednisolone | Corticosteroid | Effect modifier |
